# Complete Blood Count-Derived Inflammatory Markers and C-Reactive Protein in Testicular Cancer: Diagnostic and Prognostic Utility

**DOI:** 10.3390/medsci13040274

**Published:** 2025-11-17

**Authors:** Dragoș Puia, Marius Ivănuță, Victor Mihail Cauni, Mihaela Corlade-Andrei, Cătălin Pricop

**Affiliations:** 1 Grigore T. Popa University of Medicine and Pharmacy Iasi, 700115 Iasi, Romania; dragos-puia@umfiasi.ro (D.P.); catalin.pricop@umfiasi.ro (C.P.); 2 Department of Urology, “Dr. C.I. Parhon” Clinical Hospital, 700503 Iasi, Romania; 3 Center for Morphological and Spectroscopic Analysis of Urinary Stones” Michel Daudon”, 700503 Iasi, Romania; 4 Department of Urology, Colentina Clinical Hospital, 020125 Bucharest, Romania; victorcauni@yahoo.com; 5 Emergency Care Department, “Sf. Spiridon” County University Emergency Hospital, 700111 Iaşi, Romania

**Keywords:** testicular cancer, inflammatory markers, neutrophil-to-lymphocyte ratio, C-reactive protein, prognosis, diagnosis

## Abstract

Background: Traditional tumor markers used in testicular cancer diagnosis, such as alpha-fetoprotein (AFP), beta-human chorionic gonadotropin (hCG), and lactate dehydrogenase (LDH), present limitations due to variable expression across tumor subtypes. Inflammatory markers derived from complete blood count (CBC), along with C-reactive protein (CRP), have emerged as potential adjuncts for diagnosis and prognosis. This study aimed to evaluate the diagnostic and prognostic utility of CBC-derived inflammatory indices and CRP in patients with testicular cancer. Methods: We retrospectively analyzed testicular cancer patients, assessing baseline CBC parameters, inflammatory ratios (including neutrophil-to-lymphocyte ratio [NLR], platelet-to-lymphocyte ratio [PLR], and systemic immune-inflammation index [SII]), and CRP levels. Their diagnostic accuracy was compared with classical tumor markers, while prognostic implications were assessed through survival outcomes and disease progression. Results: Inflammatory markers, particularly NLR and SII, demonstrated significant associations with tumor burden and advanced disease stage. Elevated CRP levels correlated with poorer prognostic features and worse outcomes. While classical tumor markers remained essential in diagnosis and staging, the integration of inflammatory indices provided additional discriminatory power, especially in patients with normal or equivocal AFP and hCG values. Conclusions: CBC-derived inflammatory markers and CRP represent promising, cost-effective, and easily accessible tools that complement classical tumor markers in testicular cancer. They offer both diagnostic and prognostic value, particularly in cases where traditional biomarkers are insufficient. Prospective multicenter studies are warranted to validate these findings and incorporate inflammatory indices into routine clinical algorithms for testicular cancer management.

## 1. Introduction

The diagnostic assessment of testicular cancer involves clinical examination, imaging techniques, and tumor biomarker analysis to determine the suspicion of malignancy and the extent of the disease. Biochemical tumor markers, such as alpha-fetoprotein (AFP), beta-human chorionic gonadotropin (hCG), and lactate dehydrogenase (LDH), serve as standard adjuncts in diagnosis and are incorporated into staging systems like the International Germ Cell Cancer Collaborative Group (IGCCCG) classification [1]. Classical serum markers exhibit limitations stemming from their variable expression. Specifically, alpha-fetoprotein (AFP) is not elevated in pure seminomas, while human chorionic gonadotropin (hCG) elevation occurs in only a minority of seminoma cases, resulting in false negative outcomes for some patients [2].

The evaluation of systemic inflammatory responses via complete blood count (CBC)-derived markers has increasingly been acknowledged as a supplementary tool in oncological diagnostics and prognosis. Testicular cancer, a prevalent solid malignancy among young men, is associated with emerging evidence that underscores the importance of biomarkers in disease stage stratification and predicting patient outcomes [3]. These parameters can be calculated from routine laboratory data, offering a low-cost, widely accessible method to complement conventional diagnostic and staging tools or some immunohistochemical markers such as SALL4 and OCT3/4 [4]. In testicular tumors, Imamoglu et al. have shown that certain threshold values of these markers may carry diagnostic or staging implications; for example, a neutrophil-to-lymphocyte ratio (NLR) value above 2.7 has been suggested to support diagnosis when compared to healthy individuals, while an NLR cutoff near 4.0 could differentiate advanced from early-stage disease [5].

In addition to single-ratio indicators, composite markers such as the SII integrate platelet counts into established ratios to improve prognostic accuracy. In the context of testicular cancer, elevated SII values have been associated with more advanced disease stages as defined by pathological staging criteria. These associations underscore their potential function in early risk categorization and their relevance throughout various stages of clinical management [2].

CRP is the main acute-phase reactant that directly measures systemic inflammation. On the other hand, inflammatory ratios derived from the CBC (like NLR) give a more complete picture by showing the cellular part of the host-tumor immune interaction. Both markers are intrinsically connected via the signaling cascade initiated by pro-inflammatory cytokines (e.g., IL-6), which concurrently enhance hepatic CRP production and regulate the mobilization and distribution of circulating leukocytes. Nonetheless, the NLR’s prognostic superiority in certain cancer types is believed to arise from its ability to reflect both the adverse consequences of systemic inflammation (neutrophilia) and the insufficiency of the anti-tumor adaptive immune response (lymphopenia). So, even though a high CRP level shows that there is a lot of inflammation in the body, a high NLR level gives a more detailed picture of the immune system, showing both the short-term reaction and the long-term suppression of the immune system in the case of testicular cancer. We aimed to compare the values of CBC-derived inflammatory markers and C-reactive protein (CRP) between patients with testicular cancer and a control group.

## 2. Materials and Methods

Data of patients who were diagnosed with testicular cancer who underwent radical orchiectomy within the Urology Clinic of Dr.C.I.Parhon Hospital Iasi, Romania between January 2020 and August 2025 were retrospectively collected. Radical orchiectomy was conducted in accordance with the recommended guidelines. Patients with disorders that could influence inflammatory markers, including additional malignancies, active or chronic infections, immunosuppressive diseases, systemic inflammatory conditions, the use of immunosuppressive medications, and renal or hepatic dysfunction, were excluded from the study. The control group was formed by patients treated during the same period for a benign scrotal condition, namely hydrocele. From both groups, we collected data such as age at the moment of surgery, complete blood count, C-reactive protein, serum creatinine, beta-HCG, AFP, LDH and the largest tumor dimension as measured by the pathologist. The CBC-derived inflammatory markers have been calculated as follows: NLR = neutrophil count/lymphocyte count, MLR = monocyte count/lymphocyte count, Systemic Immune-Inflammation Index (SII) = platelet/× neutrophil-to-lymphocyte ratio, Systemic Inflammation Response Index (SIRI) = (neutrophil count × monocyte count)/lymphocyte count; Aggregate Index of Systemic Inflammation (AISI) = (Neutrophil count × Monocyte count × Platelet count)/Lymphocyte count.

The Mann–Whitney U test was employed to compare each parameter between groups. Data were presented as mean ± standard deviation and median; *p* < 0.05 was accepted as statistically significant. The area under the receiver operating characteristic (ROC) curves was assessed for the markers that were significantly different between the two groups. in the detection of testicular malignancies. We also made a subgroup analysis between the main histologic types.

Approval was obtained from the hospital Ethics Committee (Approval no. 8272/1 September 2025). Statistical analysis was performed by using the Statistical Package for the Social Sciences version 26.0 (SPSS Inc., Chicago, IL, USA).

## 3. Results

A total of 42 patients diagnosed with testicular cancer and 102 individuals in the control group were initially enrolled. Within the control group, four participants had a documented history of malignant disease (three cases of prostate cancer and one case of colon cancer) and were therefore excluded. The final control cohort comprised 98 individuals.

Among patients with testicular cancer, 26 (61.9%) exhibited abnormal levels of at least one of the classical serum tumor markers (AFP, β-hCG, LDH). In this subgroup, the mean AFP concentration was 210.42 ± 101.59 ng/mL, the mean β-hCG level was 17.40 ± 14.06 mIU/mL, and the mean LDH level reached 898.65 ± 787.33 U/L, indicating a marked biochemical elevation consistent with active disease.

When comparing baseline clinical characteristics, no significant differences were observed between patients with testicular cancer and the control group regarding mean age, serum hemoglobin concentration, or serum creatinine levels (*p* > 0.05 for all comparisons). This suggests that the two groups were comparable in terms of general demographic and laboratory parameters unrelated to malignancy.

In the evaluation of systemic inflammation, no statistically significant differences were identified in CRP, MLR, or SIRI values between the two groups (*p* > 0.05). However, several composite inflammatory indices demonstrated significant associations with testicular cancer. Specifically, NLR, SII, and AISI values were significantly higher in patients with testicular cancer compared to controls (*p* < 0.05 for all). These findings support the role of immune-inflammatory responses in the biological profile of testicular cancer.

The detailed distribution of inflammatory markers and the statistical analysis of differences between the two groups are summarized in Table 1.

Histopathological evaluation revealed that 22 patients (53.38%) presented with seminomas, while the remaining 20 patients (47.62%) were diagnosed with non-seminomatous germ cell tumors. The distribution of the non-seminomatous group was as follows: embryonal carcinoma in 8 cases (19.04%), teratoma in 7 cases (16.66%), choriocarcinoma in 3 cases (7.14%), and yolk sac tumor in 2 cases (4.76%).

In the subgroup analysis comparing the two major histological categories (seminomas vs. non-seminomatous tumors), no statistically significant differences were observed in terms of mean age, maximum tumor diameter, serum CRP, NLR, MLR, or SII (*p* > 0.05 for all). These findings suggest that the baseline demographic and general inflammatory characteristics are relatively similar across the two main histological subtypes.

However, significant differences were identified in the values of SIRI and AISI between seminomatous and non-seminomatous tumors (*p* < 0.05). This indicates that these two composite inflammatory indices may better reflect the distinct biological behavior of testicular cancer subtypes. The detailed results of this comparative analysis are presented in Table 2.

To further assess the diagnostic performance of the inflammatory markers that showed significant differences between patients with testicular cancer and controls, we performed a receiver operating characteristic (ROC) curve analysis. However, the discriminatory ability of these indices was limited.

**Figure 1:**
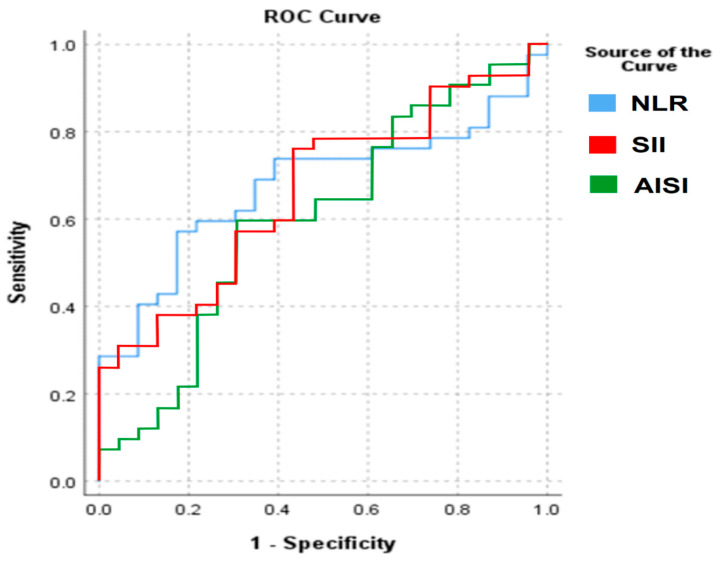
ROC analysis of the significantly different CBC-based markers.

For NLR, the area under the curve (AUC) was 0.677, while for SII the AUC was 0.666, and for AISI the AUC was 0.601. According to standard interpretative thresholds, these values indicate only poor to fair discrimination between patients with testicular cancer and control subjects. The corresponding ROC curves are presented in Figure 1.

## 4. Discussion

The relationship between inflammation and cancer development is governed by a complex array of cellular and molecular mechanisms that collectively foster a microenvironment beneficial to tumor initiation, growth, and metastasis. According to Salazar-Valdivia et al., chronic inflammatory processes serve both as initiators and promoters of carcinogenesis by perpetually stimulating proliferative signals, facilitating evasion of apoptosis, and inducing genetic instability [6]. Tumor cells can maintain this state by secreting cytokines, chemokines, and prostaglandins, which attract immune cells and support an inflammatory environment conducive to their survival [7].

CRP is an acute-phase reactant primarily produced by hepatocytes in response to pro-inflammatory cytokines, particularly interleukin-6, during systemic inflammatory activation. In oncology, according to Bleve et al., CRP serves as a surrogate indicator of systemic inflammation and functions as an active modulator of tumor biology. Persistent elevation may indicate cancer-associated inflammation driven by tumor-derived mediators, which contributes to disease manifestations and influences progression trajectories [8]. Although in our group, CRP did not vary significantly between groups, in the case of other types of urological cancers, such as renal cancer, the situation is different [9]. However, different studies suggested that CRP has potential as a predictive biomarker in testicular germ cell tumors (TGCTs) as it can reflect systemic inflammation linked to tumor presence and progression. In the context of TGCT-specific circumstances, despite the scarcity of direct, large-scale, longitudinal datasets, analogies can be inferred from smaller series and mechanistic research. According to Janicic et al., inflammatory cascades triggered by primary or metastatic lesions stimulate CRP generation and induce hematological changes, including neutrophilia and thrombocytosis [10]. Consequently, increased pre-treatment CRP may indicate both the present tumor load and the extent of the host’s systemic response that promotes disease development. The predictive interpretability of CRP is partially dependent on its integration with additional biomarkers. Dynamic alterations in CRP possess significant clinical implications. According to Yuksel et al., postoperative declines following orchiectomy may indicate the removal of the primary inflammatory source. In contrast, sustained elevation during follow-up, particularly in the absence of infection or other inflammatory conditions, may suggest residual disease or early recurrence prior to the detectable rise in traditional serum tumor markers such as alpha-fetoprotein or human chorionic gonadotropin [11].

NLR reflects the dynamic relationship between innate (neutrophils) and adaptive (lymphocytes) immune responses during illness, systemic inflammation, and stress [12]. The NLR has been investigated as a prognostic and potentially diagnostic adjunct to conventional serum tumor markers in the context of TGCTs. NLR may offer supplementary insights by reflecting systemic inflammatory changes indicative of tumor biology, rather than solely representing direct tumor secretion products. Imamoglu et al. indicate that increased pre-treatment NLR values are associated with more advanced clinical stages in TGCT patients and may independently predict poorer progression-free survival, regardless of IGCCCG classifications [5]. The biological rationale for its involvement in TGCT is rooted in the mechanistic pathways observed in other malignancies: neutrophilia serves as a marker of tumor-promoting inflammation, while lymphopenia reflects suppressed adaptive immunity. In our study, NLR was significantly higher in testicular cancer patients. According to Lesko et al., post-orchiectomy measurements frequently indicate a significant reduction in systemic inflammatory drive subsequent to the excision of the primary tumor mass. An ongoing increase in NLR during follow-up may indicate minimal residual disease or hidden metastasis prior to a significant rise in conventional markers such as AFP or hCG [13,14].

The MLR indicates the proportional connection between two leukocyte subsets, which have separate yet linked functions in antitumor immunity and tumor-promoting activities. Lymphocytes, especially cytotoxic CD8+ T cells and natural killer cells, are a pivotal component of adaptive and innate immune surveillance, adept at identifying and eliminating malignant cells via perforin- and granzyme-mediated cytolysis. Meanwhile, monocytes act as precursors to macrophage populations in the tumor microenvironment, where they frequently differentiate into alternatively activated, M2-type tumor-associated macrophages (TAMs) under the influence of interleukin-4, interleukin-13, and transforming growth factor-β secreted by both stromal and neoplastic components [15,16]. Relationships between reduced MLR and indicators of aggressive disease biology transcend stage evaluations. In TGCT cohorts, larger primary tumors and increased blood LDH levels have been associated with reduced MLR readings [17,18]. The utility of MLR is not diminished by histological subtype variation. Wang et al., indicated no statistically significant difference between seminomatous and non-seminomatous TGCT preoperative MLR values, implying that its prognostic signal is consistent across major subgroups [2]. The application of LMR in TGCT management may enhance various aspects of patient care. According to Sarejloo et al., a low ratio at presentation may indicate cases at increased risk for advanced disease prior to the completion of comprehensive imaging evaluation. In the post-orchiectomy surveillance phases, consecutive decreases in MLR, particularly when accompanied by slight increases in other inflammatory markers, may warrant earlier radiologic assessment for recurrence prior to the emergence of clinical or biochemical progression [19].

SII encapsulates various dimensions of host immune and inflammatory status in a single metric. The increase in neutrophils and platelets quantitatively supports their simultaneous elevation, a condition frequently seen in tumor-promoting inflammation, while division by lymphocytes adjusts this value based on the extent of adaptive immune suppression present [2]. Recent studies have broadened the evidence concerning the SII in testicular germ cell tumors (TGCTs), building upon previous research on simpler ratios like the NLR and PLR. High indices indicate a pro-tumoral immune environment marked by heightened innate activation, suppression of adaptive immunity, and vascular facilitation driven by thrombocytosis. According to Imamoglu et al., patients with TGCT often exhibit elevated baseline SII values compared to those with benign scrotal conditions, similar to the observed trends for NLR and PLR [5]. Determining the cut-off remains a critical methodological point in interpreting emerging data. Bleve et al. propose thresholds near 844 or 1000 for dichotomizing high versus low risk; these derive from receiver operating characteristic curve optimization, balancing sensitivity with specificity [8]. Variation exists among cohorts based on case mix, laboratory reference ranges, and exclusion criteria for concurrent inflammatory conditions. Standardization is essential for advancing from retrospective associations to routine clinical adoption. Emerging literature acknowledges limitations, including the sensitivity of hematologic counts to intercurrent illness or corticosteroid use, which can transiently skew index results independently of malignancy [20]. Future prospective trials must implement stringent exclusion or adjustment protocols to address potential confounding effects.

Our study exhibits several limitations. Notably, its retrospective and single-center design limits generalizability. The limited sample size diminishes statistical power and heightens the likelihood of a type II error. Certain inflammatory markers demonstrated limited discriminatory ability (AUC < 0.70), which restricts their clinical applicability. Furthermore, potential confounding factors, including subclinical infections or medications affecting blood counts, may not have been entirely excluded. The lack of prospective validation diminishes the robustness of the conclusions drawn.

## 5. Conclusions

Recent advances in the evaluation of systemic inflammatory responses using complete blood count–derived indices and C-reactive protein have opened new avenues for improving the diagnostic and prognostic assessment of testicular germ cell tumors. When considered alongside traditional serum tumor markers (AFP, β-hCG, and LDH), these inflammatory markers contribute to a more comprehensive characterization of tumor burden and biological aggressiveness.

Future research should focus on prospective validation of these findings, the integration of inflammatory indices, and the classic prognostic serum markers (AFP and hCG). Additional avenues include novel molecular biomarkers and the development of composite prognostic models that combine biochemical, cellular, and molecular parameters. Such integrative approaches hold the potential to optimize clinical decision making, refine risk stratification, and ultimately improve long-term outcomes in this predominantly young patient population.

## Figures and Tables

**Table 1:** Comparison of characteristics between the two groups.

Parameter	Testicular Cancer (*n* = 42)	Control (*n* = 98)	*p*
Mean age (years), SD	40.76 (±14.44)	43.83 (±12.44)	0.20
Serum hemoglobin (g/dL)	14.63 (±1.68)	14.92 (±1.07)	0.22
Serum creatinine mg/dL	0.92 (±0.18)	0.98 (±0.21)	0.37
CRP (mg/L)	2.98 (±2.84)	2.53 (±2.02)	0.25
NLR	3.08 (±2.78)	1.95 (±0.57)	0.029
MLR	0.34 (±0.19)	0.28 (±0.08)	0.09
SII	839.39 (±796.51)	479.73 (±173.48)	0.039
SIRI	2.02 (±1.8)	1.26 (±0.7)	0.10
AISI	634.13 (±484.48)	312.66 (±194.93)	0.042

**Table 2:** Comparison of characteristics between the two main histological types.

Parameter	Seminomas (*n* = 22)	Non-Seminomas (*n* = 20)	*p*
Mean age (years), SD	40.45 (±11.08)	41.05 (±17.20)	0.44
Tumor diameter (cm), SD	4.98 (±2.17)	4.16 (±3.35)	0.17
CRP (mg/L)	3.26 (±2.30)	2.69 (±3.33)	0.26
NLR	3.68 (±3.67)	2.48 (±1.28)	0.08
MLR	0.36 (±0.21)	0.32 (±0.16)	0.24
SII	957.5 (±1107.29)	635.52 (±404.78)	0.10
SIRI	2.69 (±2.18)	1.42 (±0.9)	0.02
AISI	846.36 (±604.1)	364.81 (±279.21)	0.01

## Data Availability

The original contributions presented in this study are included in the article. Further inquiries can be directed to the corresponding author(s).

## Abbreviations

The following abbreviations are used in this manuscript:

AFP	alpha-fetoprotein
LDH	lactate dehydrogenase
CBC	complete blood count
CRP	C-reactive protein
NLR	neutrophil-to-lymphocyte ratio
PLR	platelet-to-lymphocyte ratio
SII	systemic immune-inflammation index
ROC	receiver operating characteristic
AUC	area under curve
